# Holiday Reminiscences of Hawaii

**Published:** 1911-09

**Authors:** John C. Warbrick

**Affiliations:** Chicago


### TEXAS MEDICAL JOURNAL.

Established July, 1885

F. E. DANIEL, M. D., - - - - - - Editor, Publisher and Proprietor

Published Monthly.—Subscription $1.00 a Year

VOL. XXVII. AUSTIN. SEPTEMBER, 1911. No. 3.

The publisher is not responsible for the views of the contributors.

### Original Articles.

For Texas Medical Journal.

### Holiday Reminiscences of Hawaii.

BY JOHN C. WARBRICK, M. D., CHICAGO.

It was at G. W. McDougall's coffee plantation, a few miles up Mount Hualalai, from Kailua, North Koua, the Hawaiian Islands, September 15, 1894, when word was brought in a hurry by a native courier that a woman was dying at Kahoniki, and immediate help was needed. "Not a moment is to be lost," said McDougall. "If you decide to go, I will join you as interpreter, and we may get there in time to do something for the woman." No sooner said than accepted, while two fast mountain horses were soon waiting at the door. Losing no time, we mounted at once and were then off at full speed along the narrow path leading across Mount Hualalai in the direction of Kahoniki, having about four miles to go. McDougall being a good rider and knowing the route well, led all the way at a good pace. Up and down small hills on the mountain, around sharp corners, across ditches, over rocks and through bushes we pushed our way without any stops.

Having set a fast pace all the way and being well in the lead, McDougall at times called out. "Come on. No time is to be lost. What is the matter?" Any one not much accustomed to riding then to mount one of these bony mountain animals which are much the same as the cowboys use in Texas for a few miles' ride at fast speed along an unknown path would understand what the matter was.

In a good deal less than an hour we reached Kahoniki, drawing up at the door of an old primitive Hawaiian hut of wooden logs, straw roof and with only the floor as a seat. Off of our horses, we were soon inside the old wooden shack, when at a glance it was quite evident what the trouble was.

A large Kanaka wahine was found lying on the floor on her back gasping for breath, having been that way for about five hours. She had been trying to deliver herself of a child and had nearly succeeded in all but the head, which for some reason or other could not be got out, although trying for a long time and tugging at it as she had the strength, but making no headway. The rest of the child's body was outside the vulva lying on the floor between her legs, being quite cold and dead, while the woman was getting weaker all the time from the useless efforts at straining to deliver the head, while the pulse was feeble. She was a multiparæ about 35 years old, and had easily delivered herself of several other children without any assistance whatever; rather a common occurrence among the native women of Hawaii and only in extreme cases do they ask for any medical assistance, on account of their superstitious ideas.

To relieve the weakening woman as soon as possible was the first thing to be done. So with coat off and sleeves rolled up McDougall said go ahead as quick as you can. Starting to take hold of the child's body to release the head, suddenly Kanaka superstition somewhat strong on the part of the husband, he refused any assistance, saying he would let his wahine (wife) die rather than have any aid, for fear of some ill omen. With much argument and forcible language about the matter for a time on the part of McDougall as interpreter, it was however of no use, and rather a surprise. Leaving the shack, we went a short distance farther up the mountain to a frame house where we sat on the veranda for a time to talk the matter over and decide what to do at McDougall's suggestion.

Returning to the shack, McDougall said as a last resort he would use the most forcible (Kanaka) native language he could to induce the husband to have something done at once or it would be too late, for we had in a short time to return to the plantation. This had desired result; so the husband said to go ahead and do what could be done for his wahine's condition to save her if possible. Beginning at once to try and deliver the child's head, the body was manipulated in different ways, but no use; then traction on the legs was tried and at the same time pushing on the head from the abdomen, but this method failed to release the head any and the same of traction on the cord. Without losing any time, washing my hands in plain water as the only antiseptic precaution taken for this was the only liquid near, passed my right hand into the uterus and found the head was movable, while a loop of cord was wound around the neck. The head was large, the bones compressible and the sutures could be readily felt. Trying to compress the bones together enough to relieve the head was of no avail. What to do was rather a puzzle. No time could be lost; some method had to be found. A small penknife with blades dull and a little rusty was the only available instrument at hand for use. Washing and wiping the blades, then with a small blade open, guarding the knife beneath the palmar surface of right hand and the blade guarded and directed by the index finger, passed it into the uterus and felt for a soft spot on the head. This was not hard to find; so pushed the point of the blade through it, when a good deal of fluid came away. Making a larger opening and compressing the bones with my hand a good deal more fluid came away, thus reducing its size, while by using traction on the body the head was gradually delivered, being large in size and a hydrocephalus. Soon afterwards the placenta came away and the woman made a good recovery, being able to get around in a few days, as is the case with most of them after a confinement. In fact, it is somewhat remarkable to think that a great many of the Hawaiian women can be confined one day and be able to get about the next without any bother.

Early the next morning word came to the house that a Japanese woman was ill and needed immediate attention. As she lived only a short distance away, McDougall said we had better go at once and see what could be done for her, and he would act as interpreter. When we got to the place it was found the woman was five months pregnant and had injured herself by a fall, feeling weak as a result of it. Upon a careful examination, it was soon evident a condition was present not easy to handle; namely, an impacted transverse presentation with prolapse of the right arm and the umbilical cord, part of which was outside the vulva. Trying to keep the cord up out of the way could not be done and it was almost impossible to move the arm in any direction. Getting the head down or even moved at all could not be done, the body was so firmly wedged in it could hardly be moved. The child was dead, and the only way it could be got out was by lacerating or cutting it by pieces. The husband gave his consent to any way that would relieve his wife and prolong her life. The right arm had to be cut with a knife and be wrenched from the body and the same of the head, while the rest of the body came away all right, and the woman made a good recovery, as there was no hemorrhage.

